# Mutant p53 Drives Cancer by Subverting Multiple Tumor Suppression Pathways

**DOI:** 10.3389/fonc.2016.00012

**Published:** 2016-01-27

**Authors:** Sue Haupt, Dinesh Raghu, Ygal Haupt

**Affiliations:** ^1^Tumour Suppression Laboratory, Peter MacCallum Cancer Centre, Melbourne, VIC, Australia; ^2^Department of Pathology, The University of Melbourne, Parkville, VIC, Australia; ^3^Sir Peter MacCallum Department of Oncology, The University of Melbourne, Parkville, VIC, Australia; ^4^Department of Biochemistry and Molecular Biology, Monash University, Clayton, VIC, Australia

**Keywords:** p53 mutations, gain of function, metabolism, cell cycle, transcriptional regulation

## Abstract

The tumor suppressor p53 normally acts as a brake to halt damaged cells from perpetrating their genetic errors into future generations. If p53 is disrupted by mutation, it may not only lose these corrective powers, but counterproductively acquire new capacities that drive cancer. A newly emerging manner in which mutant p53 executes its cancer promoting functions is by harnessing key proteins, which normally partner with its wild type, tumor-inhibiting counterpart. In association with the subverted activities of these protein partners, mutant p53 is empowered to act across multiple fundamental cellular pathways (regulating cell division and metabolism) and corrupt them to become cancer promoting.

## Introduction

Reliance on the tumor suppressive capacity of p53 is profoundly emphasized by its near universal malfunction in all cancers. P53 is the most altered gene in cancer. More than 50% of human cancers are afflicted with a p53 mutation. Severe consequences of p53 mutation include the failure to protect against cancer stimuli, compounded by the acquisition of new cancer promoting, “neomorphic” properties, referred to as “Gain of function” (GOF), covered by other reviews in this series [reviewed in Ref. (1)].

**Figure 1:**
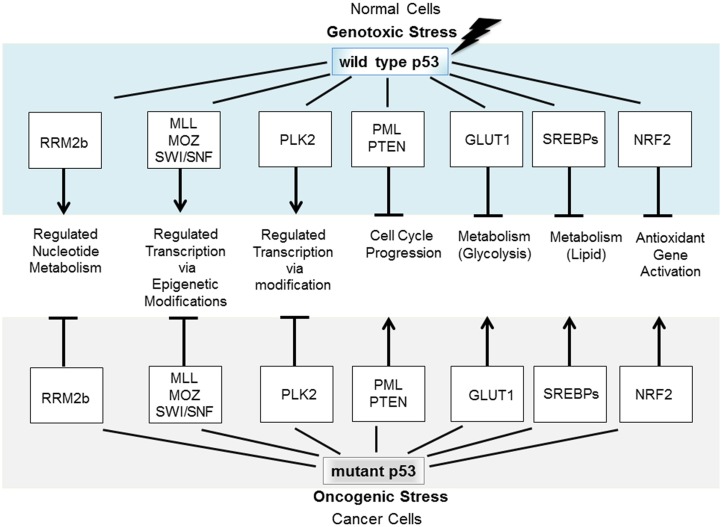
**Wt p53 is induced to accumulate in response to stress to regulate fundamental cellular processes that protect against tumorigenesis**. If p53 becomes mutated, it not only loses these tumor-protecting capacities but also may gain new functions through coercion of partner molecular partners normally engaged by wt p53.

A particularly sinister GOF constitutes the subversion by mutant p53, of molecular partners of wild type (wt) p53, and this strategy forms the focus of this review. Specifically, mutant p53 conscripts proteins that normally partner with wt p53. This new association divests them of their anticancer activities and in place, they are corrupted to act as promoters of tumorigenesis [e.g., Ref. (2)]. A number of fundamental cellular functions that are normally tumor suppressive under the directive of wt p53 become severely derailed under the influence of mutant p53 to promote cancer. Mutant p53 deregulates normally tightly controlled fundamental processes (including control of the mitotic cell cycle, glycolysis, nucleic acid, and lipid synthesis) to promote deregulated, proliferative cancer cell growth (Figure 1). Identifying the nature and the regulation of this mutant p53, GOF predicts therapeutic avenues for reining-in the impact of mutant p53 and fighting cancer.

## Subversion of Cell Cycle Regulation

### Promyelocytic Leukemia

Proper cell cycle regulation is vital for normal cell function. Equally critical is the capacity to sense DNA damage and to interrupt the cycle to instigate repair or eliminate cells with irreparable damage, as appropriate. Wt p53 is a key dictator of cellular fate in response to DNA damage resulting from cellular stresses. Partnership with the tumor suppressor promyelocytic leukemia (PML) protein facilitates p53 stress responses. Specifically, wt p53 stabilization and activation in response to stress is promoted by PML, through temporal co-recruitment of post-translational modifiers of p53 [kinases: CK1 (3), CK2 (4), HIPK2 (5); acetylases: CBP/p300 (6); MOZ (7)], to functional service depots, known as “PML nuclear bodies” (PML-NBs). PML-NBs facilitate the addition of post-translational modifications to p53, which relieve it from its normally labile state. Stabilized wt p53 accumulates, halts cell cycle progression, and initiates molecular responses to either repair DNA or direct the execution of incurable cells. PML in turn is a direct target of wt p53 transcriptional activation, which defines a positive regulatory loop (8). Further, PML-NBs associate with sites of active transcription and appear to facilitate gene expression (9). PML loss alone does not cause cancer [at least in mice (10)]; however, interference with its function may promote cancer, as consistent with its discovery in acute PML, where PML is fused with RAR-alpha to generate the oncogenic PML–RAR-alpha (11).

Significantly, mutant p53 enslavement of PML defines a paradigm for mutant p53 disruption of tumor suppressive partners of wt p53. We identified that when p53 is mutated in cancer cells, its association with PML is constitutive, unlike the transient association with its wt p53 counterpart in response to stress. Importantly, PML facilitates mutant p53 to aberrantly transcribe targets in the context of hijacked transcription factor NF-Y [(2), building on foundational NF-Y studies (12)].

More explicitly, wt p53 is a transcription factor that regulates its target genes (to control DNA repair, growth, and metabolic cascades), through direct engagement of its responsive elements. In stark contrast, mutant p53 is unable to directly engage these specific elements, but rather anchors onto other transcription factors and interferes with their transcription [including NF-Y (12)]. One transcriptional target of mutant p53 in association with NF-Y and PML is CDC25C, which triggers entry into mitosis (counteracting wt p53 activated growth arrest). Consistently, mutant p53 cancer cells may become growth dependent on PML, to the point where PML depletion leads to growth inhibition (2). Paradoxically, the capacity of PML to promote wt p53 as a tumor suppressor in healthy cells redefines PML as “oncogenic” when associated with mutant p53 in cancer cells [review in Ref. (13)].

**Figure 2:**
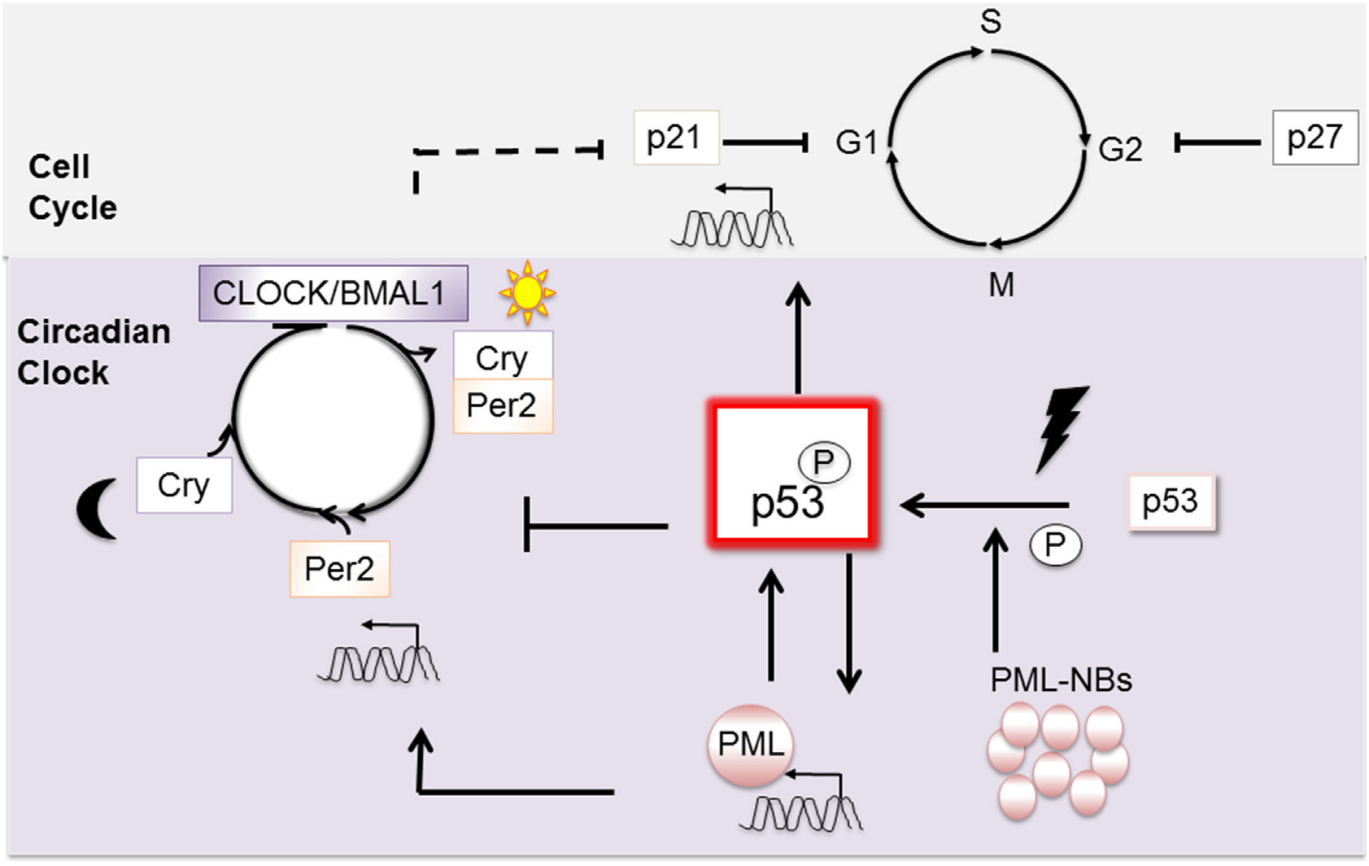
**Wt p53 is a pivotal point of connection between the mitotic cell cycle and the circadian clock**. P53 activation is promoted by its transcriptional target PML. Once activated, wt p53 intervenes in the cell cycle through expression of its target gene, the checkpoint inhibitor p21. Upon stimulation, wt p53 can also intervene to affect the circadian clock. In contrast, when p53 is mutated, its interaction with PML becomes constitutive. Cancer is exacerbated when Per is mutated on a background of p53 mutation.

At a higher level, cell cycle control is coordinated by the Circadian clock (14), and wt p53 defines a unique point of convergence between these two fundamental vital cellular regulatory systems. The Circadian clock is subject both to wt p53 (15) and PML (16) regulation and in turn regulates important cell cycle genes, including p21, independently of p53 (17) (Figure 2). While disruption of the diurnal periods of ~24 h appears insufficient alone to cause cancer, new findings suggest that it can exacerbate cancer progression [reviewed in Ref. (14)].

At a molecular level, the clock is comprised of at least nine interplaying proteins, and we will discuss only those pertinent to this review. The clock is positively activated in a cyclic fashion through the combined activities of the two transcription factors: CLOCK and BMAL1 (Figure 2). As heterodimers, they engage E-Box motifs in the promoters of their target genes and induce transcription. Important transcriptional target genes, Per and Cry, and their protein products relocate to the nucleus and negatively regulate CLOCK and BMAL1: forming a negative feedback loop. To restart the cycle, a stimulus such as light (or pertinently to our discussion DNA damage) must prompt elimination of Per and Cry, which is mediated through proteolysis [reviewed in Ref. (18)].

Wt p53 controls the clock through negative regulation of Per2 expression (Figure 2). Mechanistically, wt p53 competes for a promoter region of Per2 normally occupied by activating CLOCK/BMAL1 (15). In normal healthy cells, p53 levels oscillate temporally and Per2 levels inversely correspond. In cells undergoing stress, wt p53 accumulation inhibits Per2 transcription. On a background of mutant p53, cancer is exacerbated by mutation of either the clock regulatory gene Per2 (19), or PML loss (20). The capacity of PML to function as an upstream regulator of Per2 is consistent with a common regulatory pathway (16). In sum, interplaying regulatory loops between p53, PML, the circadian clock, and the cell cycle are emerging, and their disruption has been linked to cancer in mouse models (19, 20). Links to human cancers are also emerging, with the possibility of sleep hormone therapies being trialed [i.e., melatonin (21)].

### Phosphatase and Tensin homolog

Phosphatase and Tensin homolog (PTEN) is also a vital cell cycle regulator that has achieved its reputation as a tumor suppressor in the context of wild type (wt) p53. Pten curbs cell cycle progression and cell survival by suppressing PI3K–AKT/PKB cell survival pathway (22). PTEN functions as a tumor suppressor by stabilizing p53 protein in an Mdm2-dependent and/or -independent mechanism. (23). PTEN also increases the transcriptional activity of wt p53 through physical interaction (24). Reciprocally, wt p53 increases the transcription of PTEN by binding to the promoter of PTEN (25) and forming a feedback loop. These mutual relationships between PTEN and p53 promote tumor suppression.

In the context of mutant p53, in a diametrically opposing function, Pten promotes tumor growth (24). PTEN, in a comparable manner to PML, becomes oncogenic in cells expressing mutant p53 (26). PTEN stabilizes mutant p53 protein by inhibiting Mdm2-mediated degradation, which results in the inhibition of cell death and also in enhancement of cell proliferation (24). Additionally, PTEN increases the transcriptional activity of the mutant p53/acetylase CBP/NF-Y complex. This complex activates the transcription of c-Myc and Bcl-XL, which promotes cell survival and proliferation (26).

### Polo-Like kinase-2

Polo-like kinase-2 (PLK2) is also a wt p53 target that contributes to cell cycle control. PLK2 is transcriptionally induced by wt p53 in response to the stress of DNA damage (27). PLK2 in a wt p53 setting is tumor suppressive, as engagement of p53 response elements in the promoter of PLK2 induces cell cycle arrest at the G2 checkpoint. In contrast, in a mutant p53 context, PLK2 functions as an oncogene. Distinct, indirect interaction between mutant p53 and PLK2, mediated through the conscription of the transcription factor NF-Y (to the CCAAT box promoter sequences), increases cell proliferation. A reinforcing feed back loop is created by PLK2 in turn phosphorylating mutant p53 on a site not phosphorylated on wt p53. Phosphorylated mutant p53 interacts more efficiently with p300 and promotes transcriptional activities of cell cycle activators (28). This feedback loop involving PLK2 defines a prototype cycle of reinforcement of mutant p53 GOF (29).

## Diversion of Fundamental Cellular Pathways

Rapid cell proliferation inherent in cancer growth is utterly dependent on the ready supply of “molecular building blocks.” Recent studies have identified that fundamental metabolic processes normally regulated by wt p53 are extensively disrupted by mutant p53 to facilitate the supply of these necessities.

### Nucleotide Metabolism

#### RRM2b

Proper repair of DNA damage is orchestrated by wt p53, which not only temporally halts cell cycle progression to allow repair, but also actively facilitates the supply of constituents for the repair. Specifically, in response to DNA damage, wt p53 transcriptionally activates the small subunit of the ribonucleotide reductase (RRM2b) in a temporary manner, to facilitate the catalytic conversion of ribonucleoside diphosphates to deoxyribonucleoside diphosphates, which is an essential step for DNA synthesis. In contrast, when p53 is mutated, it constitutively upregulates RRM2b expression. Importantly, the mechanism of transcriptional activation of RRM2b is dependent on the status of p53: where wt p53 engages its REs in the intronic region and in contrast mutant p53 localizes to the promoter. Further, it has emerged that mutant p53 transcriptionally drives additional nucleotide metabolic genes, both in the salvage and new synthesis pathways, through co-recruitment with the transcription factor ETS2, to its target gene promoters. ETS2 engagement by mutant p53 is a recurring theme, as we discuss below for epigenetic regulation. Overall, mutant p53 upregulates nucleotide biosynthesis, which contributes to meeting the voracious demands of rapidly proliferating and invading cancers (30).

### Glucose Metabolism

#### Glucose Transporter 1

Regulated glucose metabolism is vital for maintaining healthy, normal cell homeostasis, in contrast to the voracious consumption of glucose that feeds cancer cell proliferation and is inherent in the “Warburg effect.” Proper glucose regulation is then an important tumor suppressive capacity of wt p53. Wt p53 regulates glucose metabolism by restricting cellular glucose at three levels through (31): (1) suppression of the expression of glucose transporter 1 (GLUT1) and 4 (32); (2) transcriptional regulation of target genes, which inhibit glycolysis [TIGAR (33)] and gluconeogenesis in the liver (34); and (3) direct binding and inhibition of the rate-limiting enzyme (glucose-6-phophate dehydrogenase) in an alternative anabolic pathway [the pentose phosphate pathway (35)].

Profoundly, when p53 is mutated, not only are these points of regulating glucose metabolism lost but further glucose uptake is accentuated through a novel GOF. This disastrous mutant p53 GOF is the shunting of the glucose transporter, Glut1, to the cell membrane surface where it stokes glucose uptake by cancer cells (36). Elevated glucose levels feed into metabolic anabolism to provide the increased demand for the molecular building blocks required to support rapid cancer cell proliferation, inherent in the Warburg effect. Reciprocally, glucose maintains mutant p53 stability and promotes cancer cell growth (37), generating a positive regulatory loop.

Reliance on a mutant p53-dependent enhanced supply of glucose to foster cell proliferation defines a unique point of vulnerability in cancer cells. This appetite for glucose identifies a potential therapy target which is currently being extensively investigated [i.e., ketogenic diets (38) and repurposing of the widely used diabetic metformin (39)].

### Lipid Metabolism

#### Sterol Regulatory Element-Binding Proteins

A controlled supply of lipids is vital for regulated cell division and maintenance. Nearly every enzyme in the fatty acid and cholesterol synthesis are subject to regulation by the transcription factor of sterol regulatory element-binding proteins [SREBPs (40)]. Specifically, SREBP-1 dictates expression of lipogenic enzymes including fatty acid synthase, while SREBP-2 regulates cholesterol synthesis [reviewed in Ref. (41)]. In response to stress, consistent with halting cell division, wt p53 restrains lipid accumulation by inhibiting expression of the transcription factor SREBP-1, and in turn triglyceride synthesis, and lipogenic genes (41). In contrast, mutant p53 engages the SREBPs (both SREBP-1 and -2) directly. Mutant p53 is recruited to SREBP target gene promoters (although co-recruitment remains to be directly demonstrated). Mutant p53 appears to upregulate transcription of key enzymes in the sterol pathway and fatty acid biosynthesis pathway. Mutant p53 correlates with increased expression of enzymes in both the mevalonate synthesis (cholesterol) pathway and fatty acid synthesis pathways. Mutant p53 upregulation of these vital pathways is consistent with meeting increased demand for membrane lipids in rapidly proliferating cancer cells (42).

### Antioxidant Pathways

#### Nuclear Factor Erythroid-Related Factor-2

Reactive oxygen intermediates perform important cellular functions including signaling; however, they are seriously damaging to normal cells if not properly contained and are linked to cancer [review in Ref (43)]. A master redox regulator is the transcription factor, nuclear factor erythroid-related factor-2 (NRF2) (44). P53 acts as a stress-rheostat controller of Nrf2 levels. Specifically, in response to mild stress, p53 transcriptionally activates the vital cell cycle inhibitor, p21, which binds to Nrf2 and consequently relieves it from its normal restraint (45). Relocation of NRF2 from the cytoplasm to the nucleus permits it to regulate multiple antioxidant targets, where some ~200 genes have been reported (44). These include the NADH-quinone oxidoreductase1 (NQO1), which also has differential function in a wt (46) versus mutant p53 context (47) (but will not be further elaborated here). When stress insults are severe, however, p53 inhibits Nrf2 (45). This exquisite level of control is consistent with p53 instigating repair in response to mild stress insults while intervening to prevent remedial action in those that are irrevocably damaged.

A novel GOF of mutant p53 is its capacity to reduce Nrf2 protein levels (without impacting its mRNA), in response to oxidative stress. The consequence is low levels of Nrf2 target detoxifying genes and elevated levels of reactive oxygen species (ROS). Remarkably, in contrast to growth inhibition imposed on wt p53 cells subject to oxidative stress, those with mutant p53 tolerate elevated ROS, survive, and proliferate (48).

## Interference with Transcriptional Regulation

When p53 is mutated, a radical shift in transcriptional activity occurs, which is conducive to cancer promotion. An altered repertoire of transcription factor engagement is emerging for mutant p53. While mutant p53 is not able to directly engage wt p53 response elements, it may instead directly bind its wt counterpart and impose a dominant negative effect over wt p53 functions, including depriving it of capacity to regulate transcription. Mutant p53 may also engage transcription factors that wt p53 does not, including the family members p63 and p73 and disrupt their functions. More specifically, the presence of arginine at codon 72 dictates the capacity of mutant p53 to sequester p73, where mutants with proline are incapable of this inactivation (49, 50).

Beyond this negative regulation of wt p53 and its family members, mutant p53 may hijack transcription factor partners and disrupt their normal transcriptional activity (as mentioned above). Mutant p53 has been reported to engage NF-Y, NF-kappa B, SP1, E2F1, ETS1, ETS2, and SREBP. The outcome may be altered target engagement, or a change in the rate of transcription relative to a wt p53 context. These features of mutant p53 have been comprehensively reviewed recently (51), so we will concentrate on new findings.

### SWI/SNF

At a higher level, mutant p53 disruption of chromatin regulation is also now emerging. In order for wt p53 to access specific DNA responsive elements in the regulatory regions (upstream promoters or introns) of its target genes, it must coordinate with numerous chromatin regulators to expose appropriate regulatory elements and associated DNA to be transcribed (52). Wt p53 exercises this activity in the context of components of the ATP-dependent nucleosomal remodeler SWI/SNF complex (53, 54). Mutant p53 has now also been identified to engage the SWI/SNF complex. However, in contrast to wt p53, mutant p53 is unable to directly engage wt p53 DNA response elements but rather localizes to distinct gene promoters through alternative transcription factors (as mentioned above). Through this co-recruitment, the SWI/SNF complex is predicted to facilitate more than 40% of all the genes transcribed by mutant p53 [where the primary example of altered regulation is the vascular endothelial growth factor receptor 2; VEGF2, which is vital for neoangiogenesis associated with oncogenesis (55)].

### MLLs/MOZ

Mutant p53 can also alter transcriptional machinery, through distinct interactions from its wt counterpart. Wt p53 is able to physically interact through its core domain with the RNA polymerase II (POL II, large subunit) and limit target gene expression (56). In contrast, through engagement of the transcription factor ETS2 [as first demonstrated in Ref. (57)], mutant p53 is able to redirect POLII to transcribe the histone methyl transferases MLL1 and MLL2 and also acetyltransferase MOZ (58). This emphasizes the insidious capacity of mutant p53 to overpower fundamental transcriptional processes to support elevated proliferation. The newly emerging application of small molecule compounds to target chromatin regulators predicts application for cancers dependent on mutant p53. Specifically, cell growth inhibition of mutant p53 cancer has been demonstrated with prototoype inhibitors (58).

## Conclusion

Corruption of the normal interactions between wt p53 and its molecular partners appears to lie at the heart of significant tumor promoting mutant p53 GOFs. Intriguingly, p53 mutations, which eliminate its function (e.g., deletion mutations), are rare, in contrast to the frequent activating missense mutations. To an extent, which appears unequaled by any other gene, mutation of p53 confers an exceptionally wide range of fundamental new properties that promote deregulated cell growth. These findings provide new insights directing innovative and rational approaches to therapeutically targeting cancers with mutant p53, which have proven particularly resistant to treatment. The polarized functions of these key p53 partners, would also caution that p53 status be an important criteria to consider prior to adoption of therapies directed toward these targets.

## Author Contributions

SH wrote the paper and prepared the figures. DR contributed to writing the paper and to preparing a figure. YH contributed to discussion and editing the paper.

## Conflict of Interest Statement

The authors declare that the research was conducted in the absence of any commercial or financial relationships that could be construed as a potential conflict of interest.
